# Medical Journalism

**Published:** 1888-01-14

**Authors:** 


### The Hospital

A Weekly Institutional Journal of

**Science, Medicine, and Philanthropy.**

*For Hospitals, Asylums, Medical Practitioners, Students, Nurses, Families, Parishes, Congregations, and the Charitable Public.*

Vol. III. No. 68.] [January 14, 1888.

### Medical Journalism.

A curious and interesting field of speculation is suggested by the consideration of the question, what humanity would have been and what it would have accomplished without the gift of articulate speech. The higher animals we see accomplish nothing, and the lower human beings very little—the latter apparently because of their defective means of making known their ideas one to another. In civilized states a tongue is one of the most potent instruments for effecting definite ends known to man. By means of it a Lesseps has joined oceans together which nature had separated, and a Bouch has united countries divided by the stormy waves of the sea. Those men by the mere strength of their own hands could have accomplished none of these things, not even if they had lived a thousand years. Their ideas might have been ten times more brilliant than they actually were, their judgment ten times more profound, and yet without the gift of speech, or some equivalent, those ideas would have been as fruitless and that judgment as impotent as the oratory of Demosthenes to a man ignorant of Greek. Possessing the gift of speech, men of genius are able to expound, to enlighten, to convince, to rouse to enthusiasm hundreds of intelligent minds, who in turn have the will and the power to set in motion tens of thousands of willing hands; and thus engineer, capitalist, mechanic, and labourer, all working together, bridge the waves, span the continents, and make the world so small that, in due time, the inhabitants of the two hemispheres shall be to each other almost as next door neighbours. Carlyle used to say " Great is the power of silence "; he would have been the first to admit that in human affairs the power of speech is greater still.

As is the tongue to the individual, so is the newspaper to the political organisation, the religious assembly, the scientific congress, the professional association. Among a host of journals in the medical field none has a more honourable record than that which is now *par excellence* the organ of the working practitioners of this country—the *British Medical Journal*. Originally commenced in a tentative way by those who felt that they were in many respects powerless before the public because they had no means of speaking with an authoritative voice, it has continued to grow in merit and influence until at the present time it has the largest circulation of any medical journal in the English-speaking world. In Great Britain the circulation exceeds that of all the other purely medical journals put together, and is more than double that of its chief rival. It may be said with truth that the Journal is every doctor's paper. For whilst the whole number of practitioners on the Register is only twenty-two thousand, the paper has a weekly issue of nearly fifteen thousand—to be quite exact, fourteen thousand seven hundred. When it is considered how many of the twenty-two thousand names on the Register are those of aged and retired practitioners, and how many others those of partners or assistants, it is at once evident that the Journal must find a home in practically every medical man's house in the United Kingdom. Various circumstances have contributed to this rapid and striking result. To begin with, the paper put a weapon of defence and of aggression into the hands of a body of men who before had been practically defenceless in the presence of those who might be friends or enemies according to their own pleasure. Experienced men know that in this world enemies of various kinds abound, and that arms are emphatically in request, both by individuals and organisations. " The great value of fire-arms," says Carlyle, " is that they make all men equally tall."

The history of the British Medical Association is a conspicuous and perhaps an unparalleled instance of what an organisation can accomplish which has a wise head and a ready and eloquent tongue—in other words, a well-conducted newspaper—at its command. The Association was founded in 1832, and as its exponent to the profession and the public issued a yearly volume of *Transactions*. No Church can make progress which preaches one annual sermon, and especially if that sermon be long, musty, fusty, and dry, as " transactions " generally are. As a matter of fact, the British Medical Association was for many years in the painful condition of a half-dead ghost, which annually gave forth a sepulchral utterance, and then subsided for a twelvemonth. Such was the Association without a weekly journal, and such, in all probability, it would have continued to this day had it still relied upon its *Transactions*.

In 1842, exactly forty-five years ago, the *Trans-*

*actions* were voted out of date, and the *British Medical Journal* was issued as a weekly. "Small and feeble" were its early beginnings—small in every way. The sheet was about half the size of the present paper, and the pages numbered only twenty as against fifty-six, or nearly three times as many, to-day. The Journal is now by far the largest of any of its medical contemporaries, the annual volume containing about two hundred pages more than the medical publication next in importance. The progress of the Journal was for many years anything but rapid; and it is important and instructive to note that the growth of the British Medical Association exactly equalled in rapidity that of the Journal. In 1842 the Association, which had then been in existence ten years, numbered only two thousand members. The next twenty-five years—until 1867—the rate of progress was quite as deliberate, averaging little more than twenty annually; so that at that date its members did not exceed three thousand.

It is from 1867—twenty years ago—that the Association and the Journal both date their extraordinary prosperity; and that prosperity, as will be seen, has been entirely due to the Journal. In 1867 Mr. Ernest Hart, the present editor, was first appointed, and, with a brief intermission, has continued to guide the fortunes of the paper during the whole of that period. Mr. Hart entered upon his work with the determination of infusing into the Journal a much more robust and decided literary and political character. His ambition was to give to a great and learned profession a tongue—a tongue in the world of science, in the social sphere, and in the greater councils of the nation. The Journal at once became thoroughly democratic; that is, democratic as representing every individual member of the medical profession, from the Queen's physician to the humblest Poor-law doctor in the kingdom.

The effect of this policy was marvellous and unprecedented. The circulation of the Journal rose by leaps and bounds, and with it the numbers and influence of the British Medical Association. Instead of an annual increase of twenty in the issue of the paper and in the membership of the Association, the increase sprang up to 500 yearly, and has ever since continued at the same rate. Last year no fewer than 800 new members joined the Association, and during the few days that have elapsed since the present year commenced 300 have been added to it. This extraordinary growth has been due to the influence of the Journal, and to the Journal alone. The relation of cause and effect is so obvious that nobody can for a moment doubt it. The *modus operandi* is this: There is sent out half-yearly to every medical man in the United Kingdom a copy of the *British Medical Journal*. Immediately thereafter there are always from 250 to 300 applicants for admission to the British Medical Association.

The financial prosperity of the Association has been equally surprising and equally due to the astonishing success of the Journal. After all expenses of every kind connected with the production and distribution of the paper have been paid—and the cost of production is not nearly covered by the yearly guinea subscribed by each member—there is an annual surplus of more than £5,000 from advertisements. This large sum is devoted to various scientific and other public purposes in the interests of the whole medical profession. The Association has purchased the lease of the large and handsome offices at which the paper is published, and in addition to this there is now a reserve fund of more than £20,000, which has been accumulated since 1872.

The British Medical Association by means of the *British Medical Journal* has frequently brought to bear upon the Legislature a degree of pressure which has been productive of results in the highest degree advantageous to the whole profession and to the public. As is well known, it has been the powerful and successful champion of poor-law medical officers, army and navy surgeons, and of a variety of other interests which but for its advocacy would have been feeble and ineffectual. In consequence of its energetic action, no fewer than three Royal Commissions have been appointed for the purpose of dealing with pressing and important questions relating to the public health and the interests of the medical profession. By its influence also the British Medical Association has now extended to India, Ceylon, South Africa, Jamaica, and to all the colonies of the British Empire. Its circulation is by no means limited to the members of the Association. At least 1,200 of its subscribers are non-members, and these pay 25*s.* annually instead of 21*s.*, the sum paid by the members of the Association. There are also 1,200 officers in the army and navy who are regular subscribers.

Such is a brief record of the rise and progress of a professional organisation and its public exponent. As we have stated, the record is probably without parallel in the history of professional journalism. To what does the Association owe its present dignified and influential position? Solely and entirely to the able and aggressive labours of its broad and philosophic Journal.

The editors of the *British Medical Journal* have mostly been men of singular ability and discretion. They have had the wit to discern and the will to resolve that their true wisdom lay in the direction of useful service towards the medical profession in all its branches. Especially has this been so in the case of the present editor; and all the disciples of British medicine know full well that wherever good cause can be shown the *British Medical Journal* at once enters the lists as a strong, courageous, and highly-trained champion, to lead the forlorn hope or to defend him who has no defender.

We have selected this conspicuous example of medical journalism because there is not in all the world such a record of great, enduring and well-merited success as its history displays. The moral from our point of view is this, that if hospitals and charitable institutions are to develop in strength, enlightenment, efficiency, and public appreciation, they too must learn to speak with united voice, to act with united hand, and to present to the world not a disorderly assemblage of incoherent units, but the serried and ordered ranks of a trained and disciplined army.

